# Specificity and Engagement: Increasing ELSI’s Relevance to Nano–Scientists

**DOI:** 10.1007/s11569-014-0194-x

**Published:** 2014-05-22

**Authors:** Barry L. Shumpert, Amy K. Wolfe, David J. Bjornstad, Stephanie Wang, Maria Fernanda Campa

**Affiliations:** 1 Oak Ridge National Laboratory, P.O. Box 2008, Oak Ridge, TN 37831-6038 USA; 2 Higher Education Research Experiences at Oak Ridge National Laboratory Program, Oak Ridge, TN USA; 3 Bredesen Center for Interdisciplinary Research and Graduate Education, University of Tennessee, Knoxville, TN USA

**Keywords:** Emerging technologies, Ethical, legal, and social issues, Nano-ELSI literature, Nanoscience

## Abstract

Scholars studying the ethical, legal, and social issues (ELSI) associated with emerging technologies maintain the importance of considering these issues throughout the research and development cycle, even during the earliest stages of basic research. Embedding these considerations within the scientific process requires communication between ELSI scholars and the community of physical scientists who are conducting that basic research. We posit that this communication can be effective on a broad scale only if it links societal issues directly to characteristics of the emerging technology that are relevant to the physical and natural scientists involved in research and development. In this article, we examine nano-ELSI literature from 2003 to 2010 to discern the degree to which it makes these types of explicit connections. We find that, while the literature identifies a wide range of issues of societal concern, it generally does so in a non-specific manner. It neither links societal issues to particular forms or characteristics of widely divergent nanotechnologies nor to any of the many potential uses to which those nanotechnologies may be put. We believe that these kinds of specificity are essential to those engaged in nano-scale research. We also compare the literature-based findings to observations from interviews we conducted with nanoscientists and conclude that ELSI scholars should add technical- and application-related forms of specificity to their work and their writings to enhance effectiveness and impact in communicating with one important target audience—members of the nanoscale science community.

## Introduction

ELSI (ethical, legal, and social issues) studies have been a formalized area of inquiry and analysis for over a generation, grappling with value-laden issues that can arise as emerging science and technology (S&T) are applied and used within the broader society. ELSI scholars have examined the potential of such technological ‘advances’ to affect the health and wellbeing of individuals, societies, and the environment both directly and indirectly, through national, corporate, and workplace policies and practices. Historically, then, ELSI activities can be considered as companions to S&T and, when viewed from that S&T, as outward–looking–toward society. This article, however, is rooted in a different and complementary premise, namely, that ELSI also ought to be inward-looking and seek to influence the nature of the S&T being developed.

To be sure, in the relatively recent past, there has been increased attention to studying ‘up,’ that is, to study and engage with physical and biological scientists as they work [4, 9, 12, 11, 10, 6]. What distinguishes this article is its contention that ELSI analyses should become far more technology- and application-specific. Simply stated, we postulate that, to engage scientists successfully, one must speak to the particularities that matter to them. Scientists’ research may fit within broad ‘nanomaterial’ or ‘nanotechnology’ categories, as examples, but their work examines very specific nano–materials or processes, contributes to specific technologies, or targets specific applications. Such particularities matter scientifically, and clearly make a difference in considering their societal impacts. For example, single-wall carbon nanotubes are significantly more toxic than either multi-wall nanotubes or fullerenes and the dose–response profiles of these three types of carbon nanomaterials are also quite different [8]. Similarly, a nanoscientist at the Center for Nanophase Materials Sciences at Oak Ridge National Laboratory reported distinctions in medical practice in the use of iron oxide nanomaterials, where this material has been readily accepted for use in disinfecting equipment but rejected in situations where the material could come into contact with the human body, such as disinfecting a catheter site on a patient’s body.

We suggest that incorporating these forms of specificity will enhance ELSI’s effectiveness in adding value to the S&T enterprise it studies, partly because they constitute a mechanism that enables ELSI scholars to frame their findings and insights in ways that are meaningful to the S&T community. The result, we believe, would be more successful incorporation of ELSI insights into decisions regarding what scientific initiatives to fund, what materials and technologies to study, and what to do with research results.

This article makes its case by focusing on nanoscale science-related ELSI (nano-ELSI), which is but one example of the class of emerging S&T that ELSI studies have addressed. Such emerging S&T as nanotechnology, biotechnology, synthetic biology, information technologies, and convergent (nano-bio-synbio-info) technologies hold in common several attributes. They are rapidly evolving in public, private, and academic sectors; they have the potential to be applied in myriad ways; and they have the potential to revolutionize both our understandings of and ways of experiencing the world. The specificity we call for is salient in these other emerging technology realms, as well.

We first set the stage by describing the context of our work and the issues it has raised about the nature of ELSI research. Then, we report the results of a review of nano–ELSI literature from 2003–2010 and use those findings as a basis for our concluding discussion.

Our research program can be traced historically to U.S. Department of Energy (DOE) ELSI funding as part of the Human Genome Program. DOE funded us to conduct a series of investigations that systematically identify and, ideally, anticipate societal issues and considerations associated with S&T whose development is supported by the agency. Our program approaches this work by analyzing evolving choices and implications associated with emerging S&T. Our principal methodology is interviewing and otherwise interacting with scientists engaged in fundamental research on emerging technologies, a characteristic that closely connects our work with the conduct of science. Broadly, we focus on choices (faced primarily by S&T managers and researchers) associated with key decision points along the trajectory from research and development (R&D) toward use. Such choices, made ‘upstream,’ potentially affect which technologies emerge, the attributes of those technologies, and, therefore, their impacts. Thus, we analyze considerations that influence what decisions are made as reflections of societal issues, particularly when the considerations include competing or conflicting interests. We also analyze the implications of different kinds of decisions, both for R&D and for the larger society. Our overarching research addresses such questions as: what information, results, and products emerge from R&D, and what is left behind; do the ‘best’ information, results, and products typically move toward use; and could unintended consequences be avoided, if only…?

As a whole, our research seeks both to contribute to ELSI scholarship and to provide information and insights that contribute to the ability of mission–inspired science to meet its societal objectives. We focus on publicly funded S&T partly because it represents an enormous taxpayer investment—billions of dollars annually—and partly because of the importance and diversity of societal missions associated with that investment. DOE’s mission statement embodies these points: ‘The mission of the Department of Energy is to ensure America’s security and prosperity by addressing its energy, environmental, and nuclear challenges through transformative science and technology solutions’.^1^

## Characterizing Nano–ELSI Literature (2003–2010)

### Approach

As part of our research effort, we reviewed nano–ELSI literature to evaluate the extent to which it incorporates the specifics that—based on our interactions with fundamental nanoscale science researchers—we hypothesize matter to members of the scientific community engaged in nanoscience R&D. We looked at whether the literature distinguished among different nanotechnologies and materials, differentiated the different potential applications of the nanoscience, and addressed issues that could be expected to resonate with the scientists conducting the research.

Our literature review encompassed two stages. The first, conducted in 2009, examined journal articles and white papers from 2003 through early 2009. In early 2011, we updated the review to include literature published from late 2009 through 2010. Our approach was akin to a snowball sampling process. We began with an initial set of 13 publications, whose authors might have been key sources of insight—individuals who were pioneering nano–ELSI scholars, and who had received early, often substantial funding from the National Science Foundation. Review of these publications suggested additional articles and prominent scholars that would contribute to a representation of the diversity of nano–ELSI research. In this first stage, we reviewed 68 articles. The second stage of the review was conducted approximately 18 months later to discover the extent to which changes had occurred in the foci of nano–ELSI studies. We reviewed an additional 23 publications in this stage. Due to the evolving focus of our own research, we emphasized nano–bio–ELSI during this stage.

In both stages of the literature review, we noted the following characteristics of each article or document: (1) the distinct nanoscience and technology characteristics discussed; (2) the particular potential applications addressed; and (3) the specific ethical, legal, or social issues central to the publication’s arguments. We then compiled and analyzed results to characterize the extent to which the literature discussed the science, technology, and ELSI concerns at a level of specificity that we believe (based on a series of formal and informal interactions with such scientists, primarily at the Oak Ridge National Laboratory) nanoscientists would find relevant to their work. Our original intent was to assure that our own research was well–grounded in extant nano–ELSI research, to build upon rather than duplicate work seeking to identify and analyze ‘the’ societal implications of nanoscale S&T. What we found during this process prompted us to take a more careful look.

### Findings

As a set, nano–ELSI studies tend to follow the pattern of earlier ELSI inquiries in that they have numerous goals, such as engaging or educating members of the public, assessing risk perceptions or technology acceptance, informing government policy, and contributing to effective regulation. Similarly, a subset of ELSI studies seeks to understand how societal considerations influence scientists engaged in nano research and, perhaps, to suggest ways to embed such considerations more effectively into their efforts.

However, our review of 91 nano–ELSI articles, book chapters, and books published from 2003–2010 revealed a significant disconnection between the ELSI literature and the nanoscientific community. For example, most nano–ELSI literature does not distinguish among nanotechnologies—it was markedly nonspecific in regard to S&T characteristics (see Table 1). Almost 64 percent of the publications we reviewed addressed nanotechnology as a single generalized category, as though societal concerns were uniform across all the different nanomaterials and technologies under study. An additional 26 percent referred to various nanotechnologies without drawing distinctions among the numerous materials, processes, and technologies involved. It is not clear whether the vast majority of these sets of publications intended to be general across all nano–materials and –processes, or whether authors had some kind or kinds of nanoscience or technologies in mind. Only 10 percent of publications explicitly discussed ELSI concerns as they related to particular materials and technologies, such as carbon nanotubes, nanoclays, nano–oxides, nanocatalysts, nanosensors, and nano–biotechnologies. Contrary to our expectation, the tendency to address nanoscale science as a generic topic was especially pronounced in the later literature we reviewed, although it should be noted that the sample size for this stage was fairly small.

**Table 1:** Percentage of nano-ELSI articles that specify science and technology characteristics

Science and Technology Characteristics	Percentage of Articles		
	2003–Early 2009 Literature	Late 2009–2011 Literature	Combined 2003–2011
All nanotechnology	60.3	73.9	63.7
Various	30.9	13.0	26.4
Carbon nanotubes; nanoparticles	1.5	4.3	2.2
Nanoclays; nanocatalysts; magnetic nanoparticles; nanosensors	1.5	0	1.1
Nanoelectronics	1.5	0	1.1
Nano-enabled brain-machine interface	1.5	0	1.1
Nanomaterials	1.5	0	1.1
Oxides of zinc, iron, cerium and zirconium	1.5	0	1.1
Nano-biotechnologies	0	4.3	1.1
Imaging	0	4.3	1.1

We noted a similar lack of specificity in regard to the potential applications to which nanotechnology might be directed (see Table 2). Most nano–ELSI literature does not distinguish among the uses to which nanotechnologies may be put. This lack of specificity was especially apparent in the later literature (late 2009–2010). More than 81 percent of the publications in our study either mentioned in passing a suite of potential applications or aggregated all possible uses of nanotechnology into a single general category and did not identify particular applications that would give rise to ELSI concerns or how those concerns might vary among the different applications. Of the almost 19 percent of publications that did make such distinctions, the main applications addressed were environmental remediation, energy production, sensors, water quality, consumer products and health and human enhancement. This subset of publications tended to focus on ELSI topics associated with individual applications, but did not scrutinize the extent to which issues or findings may be specific to one type of application versus general across different applications.

**Table 2:** Percentage of nano-ELSI articles that specify particular

Sphere of Application	Percentage of Articles		
	2003–Early 2009 Literature	Late 2009–2011 Literature	Combined 2003–2011
General	79.4	87.0	81.3
Other: environment, energy, sensors, water	5.9	4.3	5.5
Commercial	4.4	0	3.3
Health	4.4	0	3.3
Human enhancement; health	2.9	0	2.2
Military	1.5	0	1.1
Food	1.5	0	1.1
Medical	0	4.3	1.1
Agriculture	0	4.3	1.1

We found that the nano–ELSI literature identified a range of societal issues that may prove important to understanding choices, implications, and tradeoffs along the pathway from research toward use (see Table 3). Nearly half of the issues fell into just three categories: public perception (17 percent of publications), governance (15 percent), and the role of ELSI (13 percent). However, publications reviewed in the earlier stage (2003–early 2009) were notable in their emphasis on the role of ELSI (19 percent), which may reflect a sorting-out period as ELSI scholars considered how best to study the emerging technology and where their contributions lie. Other societal issues addressed in the literature included equity, risk, health impacts, environmental effects, and intellectual property considerations.

**Table 3:** Percentage of nano-ELSI sorted by particular issues of societal concern^*^

Issues of Societal Concern	Percentage of Articles		
	2003–Early 2009 Literature	Late 2009–2011 Literature	Combined 2003–2011
Role of ELSI	19.1	10.5	12.6
Perception	17.6	28.9	17.0
Equity	16.2	0	8.2
Governance	14.7	26.3	14.8
Other	8.8	2.6	5.2
Work hazard	7.4	2.6	4.4
Health	7.4	5.3	5.2
Risk	7.4	10.5	6.7
Structural	7.4	0	3.7
Dynamics	7.4	0	3.7
Intellectual property	7.4	2.6	4.4
Environmental	5.9	5.3	4.4
Unknown effects	4.4	0	2.2
Human enhancement	2.9	0	1.5
Legal	2.9	0	1.5
Privacy	2.9	2.6	2.2
Education	2.9	2.6	2.2

^*^Note that individual articles often emphasize multiple issues of societal concern.

The results of our literature review complements those reported by Shapira, et al. [13]. Shapira and colleagues examined citations in nano–ELSI literature up to the year 2007 for evidence of the emergence of a self–referential core of social science literature surrounding nanotechnology. That study found that early nano–ELSI journal articles primarily cited studies written by physical scientists and engineers (i.e., the nanoscientists themselves) and ascribed this phenomenon to ‘the lack of a social science literature specific to the emerging technology in its early phases.’ Shapira and colleagues found that this situation began to change in 2005, when public and governmental support for social science related to nanotechnologies increased. In this later literature (2005–2007), they found ‘stronger development and integration of social science literature’ relating to nanotechnology, providing ‘an interdisciplinary and cross-cutting knowledge base that is accessible to, and sourced by, social scientists writing about the societal implications of nanotechnology.’ We suggest that the evolution in nano-ELSI studies from early speculation about possible societal benefits and dangers to more developed social science methodologies, as reported by Shapira, et al., should be continued by linking the social science findings back to the nanoscientific community in ways that are relevant to the decisions they make.

Our literature review also connects with some more methodologically oriented literature. For example, a mechanism known as “interactional expertise” may prove useful for gaining technical knowledge and thereby promoting engagement with scientists. Interactional expertise focuses more on the acquisition of expertise through the process of engagement than on the purpose or impacts of that engagement—the term is defined as “the ability to master the language of a specialist domain in the absence of practical competence” [1]. Although the concept did not focus on nanotechnology originally, it can be applied to nano-ELSI scholarship. Collins and Evans proposed that “the transition to interactional expertise is accomplished, crucially, by engaging in conversations with the experts. Interactional expertise is slowly gained with more and more discussion on the science…where interactional expertise is being acquired, there will be a progression from ‘interview’ to ‘discussion’ to ‘conversation’ as more and more of the science is understood… Above all, with interactional expertise, conversation about technical matters has a normal lively tone and neither party is bored.” Another venue to gain interactional expertise is through the formation of “trading zones,” where scientists from different disciplines work together and collaborate to achieve a common goal, “some members of the trading zone [might] become sufficiently interested in the others’ work to want to understand more about it. If they pursue this ambition, it is possible that they will develop interactional expertise in one of the other disciplines” [2]. Interactional expertise may be an effective approach for ELSI scholars to adopt in becoming sensitized to the different characteristics and properties of the nanotechnology in question, thereby enhancing their ability to make their work more relevant (and easily incorporated) into the conduct of nanoscale science research.

Interactional expertise also may be gained through ethnographic approaches, such as undertaken by scholars such as Fisher [4] or Johansson [9] in laboratory settings. And, more recent publications, such the articles collected in Doom et al. [3] delineate varied engagement methods for multiple convergent technologies, including nanoscale science. In most of these cases, although the backdrops and points of reference may be technical and laboratory- or even application-specific, analyses have not tended to distinguish how the practices or issues they observed are technology- or application-specific.

## Discussion: The Missing Link and Its Implications

In many ways, this essay is motivated by a desire for ELSI scholarship to make substantive contributions to, and to be valued by, the physical scientific community. To achieve those ends, it seems reasonable to ask what questions, analyses, and insights might resonate with nanoscientists and those who manage or fund those scientists. What should members of this scientific community draw from nano-ELSI literature? It is in this vein that we have cast a self-critical eye at the published products of ELSI research.

The ELSI literature we reviewed describes a range of societal concerns associated with the emergence of nanotechnology. In so doing, it helps identify a variety of considerations that may be important to understanding choices, implications, and tradeoffs along the pathway from R&D to use. However, there is what we consider to be a critical missing link. The nano-ELSI literature largely does not tie these various concerns to particular aspects of the S&T or the uses to which they may be put. We believe that this lack of specificity impairs the ability of ELSI scholars to communicate effectively with at least one key target audience: the nanoscience community. Perhaps, in practice, the outputs of ELSI research are oriented more toward other ELSI scholars than toward the nanoscience community. It is difficult to discern, for instance, precisely how nano−scientists, −managers, and –funders could incorporate the insights of ELSI scholarship into choices they make about what to fund or study and what to do with the results of their research.

This conclusion is supported by a separate aspect of our inquiries in which we interviewed 17 scientists engaged in fundamental research at the DOE’s Center for Nanophase Materials Sciences (CNMS) at Oak Ridge National Laboratory. CNMS houses basic research activities—highly fundamental research that is quite removed from applied nanoscale science research. Our interview subjects ranged from science managers to principal investigators to post–docs and included both CNMS staff and outside users of the facility from academia and the private sector. Our interviews followed a semi–structured protocol that posed open–ended questions designed to explore the decisions that the scientists make as they pursue their research and the extent to which those decisions are influenced by or relate to societal concerns. Our analyses of interview data were descriptive, not evaluative; we aimed to gain insights, not to judge practices.

For the most part, interviewees’ responses revealed that societal issues did not factor into their day–to–day work. Interviewees described in detail the scientific questions they were exploring and the decisions they made in that pursuit, but did not frame their work (as distinct from broader nano–related research) in societal terms. These accomplished scientists described an intense focus on advancing the understanding of nanomaterials and nanotechnology in very specific ways that, in their view, had no immediate linkage to societal impacts. It appeared that the very language we used in posing questions about societal issues was problematic insofar as it was seen as not germane to these scientists’ research. After some pause, interviewees typically identified a single societal concern, such as public perception or health or environmental effects, in answer to the question we posed. Note that these issues overlap with issues that are the subject of much nano–ELSI scholarship–and hint at the opportunity for building connections between ELSI research and the nanoscience community.

Our inquiries were open–ended, and we did not prompt respondents about additional issues of societal concern or ask them about an array of potential societal issues. Nevertheless, interviewees generally did not expand their answers to encompass additional societal issues, whether within or beyond the wider range of concerns raised by ELSI scholars. Most often the scientists stated that societal concerns did not arise in their specific basic, laboratory–contained research and would be addressed by others as the outcomes of their work proceeded toward commercialization and use. Social scientists studying emerging technologies counter that decisions made during basic research may embed assumptions, concepts, and approaches that turn out to have societal implications as the technology proceeds toward use [5, 14, 7, 15]. This line of reasoning holds that a consideration of societal concerns at this early stage can facilitate beneficial outcomes and possibly avoid the surprise of negative ones.

Yet, our interactions with basic nanoscientists together with the findings from the literature reported here lead us to ponder what steps ELSI scholars might take to connect with and influence the broad nanoscience community (in contrast with individual scientists, laboratories, or research institutions). We posit that connections with members of the nanoscience community would be more readily achieved and maintained if ELSI scholars add specificity about technology attributes or potential applications to their work. Stated more boldly, ELSI scholars must be able to discuss the specific science, technology, and application characteristics that are relevant to members of the nanoscience community if they are to understand and influence the role societal considerations play in nano–related R&D. The matter of influencing the nanoscience community as a whole is even more difficult to address. It is a truism that awareness does not automatically translate into action. Related, the same awareness or knowledge does not automatically lead to the same, singular action. What is it, exactly, that we ELSI scholars want nano–scientists,–managers, or–funders to do as a result of our scholarship? We invite a dialog on this question.

## Footnotes

1 U.S. Department of Energy, Strategic Plan, May 2011. DOE/CF-0067, http://energy.gov/node/268165. See also http://energy.gov/about-us.
